# Using Geographic Information Systems to Highlight Diabetes Prevention Program Expansion Areas in Pennsylvania

**DOI:** 10.5888/pcd16.180493

**Published:** 2019-04-04

**Authors:** Brian Zepka, Mohammad Anis, Jennifer D. Keith, Duane Barksdale, Camelia Rivera

**Affiliations:** 1 Public Health Management Corporation, Research and Evaluation Group, Philadelphia, Pennsylvania; 2 Pennsylvania Department of Health, Bureau of Health Promotion and Risk Reduction, Harrisburg, Pennsylvania

**Figure:**
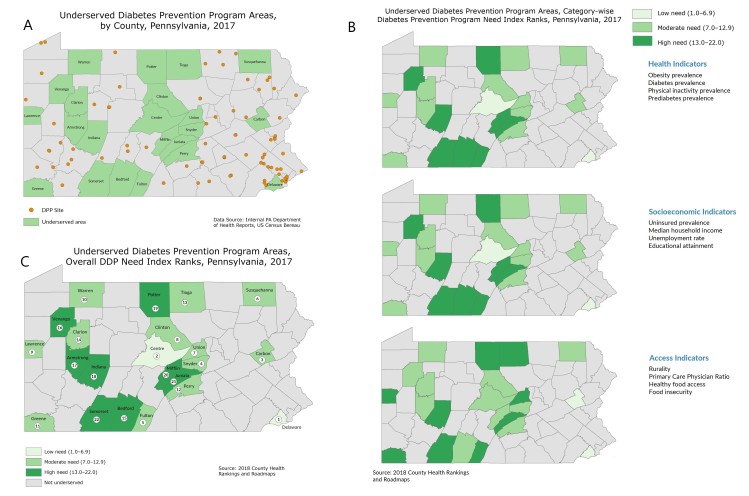
Map A shows underserved DPP areas, counties that do not have any CDC-recognized DPPs and have a population of 10,000 or more, in Pennsylvania. Map B shows the CDNIRs for each underserved county within Pennsylvania in 3 risk factor categories; health, socioeconomic, and access indicators. Numbers indicate ranking on 3 hierarchical tiers according to need for DPP: low (range, 1.0–6.9), moderate (range, 7.0–12.9), and high (range, 13.0–22.0) CDNIRs are an average of the county ranks for each indicator in the 3 categories. Map C shows the ODNIRs for the 22 underserved areas. ODNIR is a weighted average of 3 CDNIRs: health, socioeconomic, and access indicators. Abbreviations: CDC, Centers for Disease Control and Prevention; CDNIRs, Category-wise DPP Need Index Ranks; DPP, Diabetes Prevention Program; ONDIRs, Overall DPP Need Index Ranks; PADOH, Pennsylvania Department of Health.

## Background

The Diabetes Prevention Program (DPP) is a lifestyle change program recognized by the Centers for Disease Control and Prevention that is intended to prevent patients diagnosed with prediabetes from developing type 2 diabetes. The Public Health Management Corporation’s Research and Evaluation Group (R&E Group), the external evaluation partner for the Pennsylvania Department of Health’s (PADOH’s) DPP initiative, conducted an analysis to identify counties with no in-county access or limited access to sites offering DPP classes (underserved) and their relative need. R&E Group identified 22 underserved counties in Pennsylvania, a state in which diabetes is a leading cause of death. Thus, increasing access to evidence-based type 2 diabetes interventions is a priority for PADOH. To effectively prioritize DPP expansion efforts, it is important to examine resource allocation and program accessibility across the Commonwealth.

## Methods 

R&E Group produced an index to rank underserved counties on the basis of need to identify which of the 22 would benefit most from a new DPP site. This index is based on risk factors for developing type 2 diabetes and factors that influence the ability of populations to access health services. R&E Group identified 12 metrics and grouped them into 3 categories.

### Indicators

Because DPP eligibility is based on a diagnosis of prediabetes, county-level rates of diabetes and prediabetes are included as indicators of need for DPP. Prevalence of conditions considered to be risk factors for developing type 2 diabetes, including obesity and low rates of physical activity, were also included in this analysis (1).

Extensive literature indicates that low socioeconomic status is a risk factor for developing type 2 diabetes. Two socioeconomic indicators, low household income and not having a college degree, are associated with high prevalence of type 2 diabetes (2) and were included in the index. Unemployment and not having health insurance were also included, because they are typically associated with low socioeconomic status (3).

The ability for target populations to access DPP classes influences the viability of DPP sites. This index accounts for the percentage of residents living in rural areas, because these areas often experience lower access to health services than nonrural areas (4). Food insecurity and the percentage of low income populations that do not live near a grocery store were also included, because lack of consistent access to healthy food is a risk factor for developing type 2 diabetes (5). Finally, each county’s ratio of population to primary care physicians was included in this analysis to identify counties with low capacity for delivering primary care. Access to primary care is crucial to preventing chronic diseases, including type 2 diabetes (6).

### County DPP Need Index development

R&E Group used County Health Rankings data for the 22 underserved counties in each of the 12 indicators and used them to reassign each county a diabetes risk rank among the 22 counties. The new ranks were assigned based on a subset of the county’s original County Health Rankings data. The new diabetes risk-focused ranking ranged from 1 for healthiest to 22 for unhealthiest.

On the basis of this framework, a DPP Need Index (DNI) was developed by using the counties’ revised positions to determine risk for being a DPP underserved area. Counties located lower on the DNI were those that had lower levels of county-wide risks. Based on DNI rank, counties were divided into 3 hierarchical tiers according to need for DPP: low (range, 1.00–6.99), moderate (range, 7.00–12.99), and high (range, 13.00–22.00).

This methodology was applied to formulate preliminary category-wise and overall county DNI ranks. The Category-wise DPP Need Index rank (CDNIR) for a county was its average rank across all 4 metrics that make up the indicator group. The Overall DPP Need Index rank (ODNIR) was based on a weighted average ranking of the county across all 3 categories. Simple weights in each category were assigned on the basis of their direct relevance to prediabetes. Health category was assigned the highest weight of 50% in the calculation of the ODNIR on the basis of the 4 indicators (obesity, prediabetes, diabetes, and lack of physical activity) that directly affect diabetes risk in the county. High correlation among these measures indicates greater need for an intervention. Each of 2 remaining socioeconomic and access indicator categories were assigned weights of 25% in the final rank calculation as indirect indicators of diabetes risk. For example, Armstrong County, which had health, socioeconomic, and access CDNIRs of 17.00 (high), 11.25 (moderate), and 10.50 (moderate), respectively, had an unweighted ODNIR of 12.92 (moderate). However, once weights were assigned to the individual CDNIRs, the ODNIR for Armstrong County fell to 13.94 (high), because the county had a higher risk in the health category.

Data for producing this index were provided by County Health Rankings and Roadmaps (7). World Geocoder for ArcGIS (Esri) was used to locate DPP sites. Potential DPP sites were identified by using 2017 address data. Maps were produced in ArcMap version 10.4.

## Main Findings

CDNIRs and ODNIRs were used to determine counties with the highest need for DPP. Three underserved counties, Juniata, Potter, and Somerset, had high CDNIRs across all 3 indicators. They also comprised 3 of the 4 counties with the highest ODNIRs. Five other counties, Bedford, Venango, Armstrong, Indiana, and Mifflin, showed a high need for DPP on the basis of their ODNIRs. Among the original 22 counties identified as having limited access to DPP, 8 were identified as having high need for DPP infrastructure.

## Action

This series of maps highlights counties where PADOH can direct its DPP expansion efforts. Geographic visualization of DPP sites allows regional implementation partners to prioritize areas with limited program access and is a tool to engage partners in seeking expansion sites that can serve populations at high risk for developing type 2 diabetes. Our analysis focused on county data to maximize publically available data, support PADOH discussion, and align with DPP funding channels. Access across county lines is also important to explore in the future, because county boundaries are often an artificial barrier and within-county disparities in access may be missed.
